# Asymmetric Monomer Design Enables Structural Control of M(Salen)-Type Polymers

**DOI:** 10.3390/polym15051127

**Published:** 2023-02-23

**Authors:** Maria Novozhilova, Julia Polozhentseva, Mikhail Karushev

**Affiliations:** 1 Ioffe Physical-Technical Institute of the Russian Academy of Sciences (Ioffe Institute), 26 Polytekhnicheskaya Str., 194021 St. Petersburg, Russia; 2 Independent Researcher, Astana 020000, Kazakhstan

**Keywords:** asymmetric Salen-type ligand, copper polymers, electrochemical polymerization, in situ UV–vis spectroelectrochemistry

## Abstract

Conductive and electrochemically active polymers consisting of Salen-type metal complexes as building blocks are of interest for energy storage and conversion applications. Asymmetric monomer design is a powerful tool for fine-tuning the practical properties of conductive electrochemically active polymers but has never been employed for polymers of M(Salen)]. In this work, we synthesize a series of novel conducting polymers composed of a nonsymmetrical electropolymerizable copper Salen-type complex (Cu(3-MeOSal–Sal)en). We show that asymmetrical monomer design provides easy control of the coupling site via polymerization potential control. With in-situ electrochemical methods such as UV-vis-NIR (ultraviolet-visible-near infrared) spectroscopy, EQCM (electrochemical quartz crystal microbalance), and electrochemical conductivity measurements, we elucidate how the properties of these polymers are defined by chain length, order, and cross-linking. We found that the highest conductivity in the series has a polymer with the shortest chain length, which emphasizes the importance of intermolecular iterations in polymers of [M(Salen)].

## 1. Introduction

Sustainable energy storage and conversion, as well as a decrease in energy consumption, are pivotal challenges faced by modern society. To meet these challenges we need new materials that can store and convert green energy and assist in conserving energy in our everyday life. A promising class of materials that may address these challenges is conductive electrochemically active polymers [1]. Conventional examples of these materials are organic intrinsically conducting polymers with a conjugated backbone, such as polyaniline, [2], polypyrrole (PPy) [3], and poly(ethylenedioxythiophene) (PEDOT) [4]. These materials have been extensively studied and implemented as materials for electrochemical supercapacitors, batteries, electrochromic, and solar energy harvesting devices. Improving the functions and capabilities of such materials could be achieved by introducing a transitional metal center in the polymer backbone [5,6,7]. This enrichment of functions and properties arises from the properties of the metal center and from interactions between metal ions and a conjugated organic backbone.

The polymers of N_2_O_2_ Salen-type Schiff bases complexes of transition metals, [M(Salen)], are conductive and electrochemically active materials that are of interest to researchers in the field due to their advantageous properties. Specifically, polymeric [Ni(Salen)] complexes have a wide electrochemical activity window and moderate [8,9] to high (up to 100 S cm^−1^) [10] conductivity. Specific redox capacitance of [Co(Salen)] is a record-high among poly-[M(Salen)]s, with up to 182 mAh g^−1^ observed in thin polymer films [11] and scaled up to 170 mAh g^−1^ in hybrid supercapacitor electrodes [12]. Successful implementation of advantageous poly-[M(Salen)] properties in hybrid supercapacitors [10,12,13], li-ion batteries [14,15,16], electrochemical photovoltaics [17,18], and electrocatalytic [19,20,21] and sensoric [22,23] systems drive further efforts in the design and development of these materials.

Most studies in the field of poly-[M(Salen)]s are devoted to nickel-based systems. It’s safe to summarize that, in the conditions of reversible oxidative redox doping, Ni-based polymers oxidation is localized on the organic backbone, while nickel atoms effectively mediate charge transfer between organic moieties, being redox inactive [24]. In cobalt-based polymers, the metal and the organic backbone are prone to redox processes [11,25]. On the one hand, this improves capacity and catalytic activity, but on the other, decreases conductivity due to ineffective metal-mediated charge transfer between organic moieties. Generally, the same redox mechanisms are reported for oxidative chemistry of protected (unpolymerizable) [M(Salen)] complexes [26]. Copper-based polymers are less studied, especially their applications. Some researchers [27,28] postulate that oxidation of the polymer is exclusively a ligand-centered process, and it is believed that the metal center does not participate in the redox reaction. Many others believe that the process of polymer oxidation proceeds with the participation of a metal center and the formation of Cu(ΙΙΙ) [29,30]. At the same time, there is no consensus about the formation of long polymer chains or a stack structure [27,28,29,30,31]. Research of protected (unpolymerizable) Salen-type copper complexes subjected to one-electron oxidation provides different outcomes. The oxidation of [Cu(Schiff)] is metal-centered and can proceed with the formation of a low-spin diamagnetic Cu (ΙΙΙ) *3d*^8^ complex in the solid state [32]. In the solution, temperature-dependent tautomerism between Cu(III)-phenolate—Cu(II)-phenoxyl radical states [26,33] or a partially localized Cu(II)-phenoxyl radical state [34], may exist. In contrast with nickel complexes, where the oxidation process is predominantly ligand-centered when no donor species are present in the solution, the possibility of a metal-centered process [33] with the existence Cu (ΙΙΙ) [35,36] has been shown in copper complexes of the Salen-type ligands. 

The role of intermolecular forces in the polymerization mechanism and in the electrochemical behavior of poly-[M(Salen)]s is significant but not completely clear. Crystal packing of [Ni(Salen)] complex indicates the presence of weak metal—metal bonds [37] favoring dimer or infinite chain stack structures in the solid state. Copper complexes favor dimer formation in the solid state with two long copper-oxygen bonds between the two halves of the dimer due to copper coordination unsaturation [38]. Electrochemical deposition of electrochemically active deposits consisting of an [Ni(Salen)]-type monomer with extended conjugated phenazo substituent without new covalent bonds formation was revealed in [39]. A multiscale computational study found a strong intermolecular iteration of [Ni(Salen)]s in solution [40]. The research of anisotropic conductivity in polymeric complexes of nickel with Salen-type ligands support the stacked-polymer model [41]. A strong influence of intermolecular forces was also reported in many studies of poly-[M(Salen)]s [24]. At the same time, researchers postulate that polymer formation occurs by a covalent C-C bond connecting phenyl rings in monomeric fragments [42,43,44,45]. The authors confirmed, via hydrolysis, that polymerization occurs via a new C-C bond between para-positions of the monomer’s phenyl rings. The researchers demonstrated that extensive polymer hydrolysis with the breaking of imino and metal-heteroatoms bonds leads to the formation of biphenyl-4,4′-dicarbaldehyde derivatives [45]. Thus, there are two main models for the formation and structure of the polymers of [M(Salen)] complexes [29,42,43,46]: C-C bonding between phenyl moieties of monomers and intermolecular metal-phenyl or metal-metal π-stacks [47,48]. A hybrid model proposes both types of linkage [46].

An asymmetric design, e.g., Donor—Acceptor polymers, and selective polymerization of asymmetrical monomers are crucial tools for fine control of conductive electrochemically active polymers properties. These methods may lead to highly ordered structures [49]. That improves the interchain interactions between polymer chains, increases conductivity, and reduces the oxidation/reduction potential of the films [50]. A few works have been devoted to the study of asymmetric copper complexes with Salen-type Schiff bases [51,52]. No polymers of asymmetrically designed [M(Salen)] are reported so far.

**Scheme 1:**
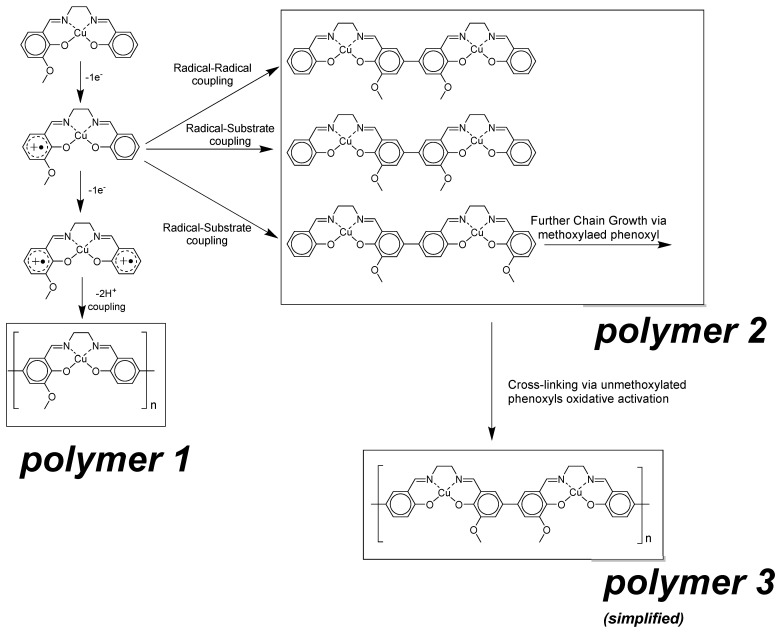
Mechanism of [Cu(3-MeOSal-Sal)en] polymerizations.

We chose the asymmetrical [Cu(3-MeOSal-Sal)en] complex (Scheme 1) with three potential redox centers: aromatic rings with and without methoxy-substituent, and a central metal atom, as a model system to probe the asymmetrical design of [M(Salen)]. We show that, due to drastic difference in redox potentials of pristine and methoxy-substituted aromatic rings, it is possible to control the C-C coupling site via simple potential control. We study three different polymeric structures using a combination of physicochemical and spectroelectrochemical methods. The dependence of the electrochemical, spectral, and structural features of polymers on the method of their formation is shown. We show that the efficiency of intermolecular charge transfer can be sufficient to impart conductivity and electroactivity to materials with an extremely low conjugated chain length, but a high degree of intermolecular interactions.

## 2. Materials and Methods

### 2.1. Chemicals

The monomer was synthesized as described in [51]. The identification of the complex was carried out using ^1^H Nuclear magnetic resonance (NMR) (Jeol 400 MHz, Japan) in DMSO-*d*_6_ and X-ray diffraction (XRD) [53]. Identification of polymer hydrolysis products were carried out using NMR (^1^H, ^13^C) in DMSO-*d*_6_.

A 0.1 mol dm^−3^ solution of tetraethylammonium tetrafluoroborate (Et_4_NBF_4_, (Sigma-Aldrich, MO, USA 99%)) in acetonitrile (CH_3_CN (anhydrous, J.T. Baker, less than 30 ppm water)) was used as supporting electrolytes. Acetonitrile was purchased from Sigma-Aldrich. Before preparing the solution, the salt was dried at 125 °C for 72 h in an inert atmosphere. The concentration of [Cu(3-MeOSal-Sal)en] in supporting electrolytes was 0.001 mol dm^−3^.

### 2.2. Electrochemistry and Electrochemical Quartz Crystal Microbalance (EQCM)

All electrochemical measurements were performed in a three-electrode cell. Polymers were synthesized in the form of thin films on conducting surfaces by electropolymerization from a solution of 0.001 mol dm^−3^ [Cu(3-MeOSal-Sal)en] and tetraethylammonium tetrafluoroborate in acetonitrile. The reference electrode was a non-aqueous Ag/Ag^+^ reference electrode (MW-1085, BASi, IN, USA) filled with a 0.005 mol dm^−3^ AgNO_3_ solution in 0.1 mol dm^−3^ Et_4_NBF_4_/CH_3_CN. The potential of this electrode was −0.30 V versus the Ag/AgCl,sat’d NaCl reference electrode. All potentials reported in this paper use the Ag/AgCl,sat’d NaCl as the reference electrode. The supporting electrolyte was a 0.1 mol dm^−3^ Et_4_NBF_4_ solution in CH_3_CN. The working electrode for cyclic voltammetry (CV) was a platinum disk (MF-2013, BASi, IN, USA, electrode area 0.02 cm^2^), that was polished with an aqueous suspension of Al_2_O_3_ (particle size 0.05 µm) prior to use.

**Polymer 1** deposition was performed in a 0.001 mol dm^−3^ monomer/acetonitrile solution with 0.1 mol dm^−3^ Et_4_NBF_4_ as the supporting electrolyte by cycling the potential of the working electrode between 0 and 1.4 V. **Polymer 2** was deposited from the same solution at constant potential of 0.85 V until the specified charge passed. **Polymer 3** was formed from **polymer 2** by cycling the potential of the working electrode between 0 and 1.4 V in monomer free supporting electrolyte.

The installation for microgravimetric studies included the complex of a QCM100 Quartz Crystal Microbalance Analog Controller, a QCM25 Crystal Oscillator (Stanford Research Systems, USA), and Bio-Logic (Science Instruments, Seyssinet-Pariset, France). Variation of the oscillation frequency of the crystal (with the natural resonance frequency of 5 MHz) due to the variation of the polymer mass on the electrode was registered automatically using a Metex MXC 1600 frequency meter (Metex Co., Seoul, Republic of Korea). We used a silicon dioxide piezoelectric crystal sprayed with a layer of platinum as a working electrode (S = 1.37 cm^2^). A counter electrode was glassy carbon plate (S = 12.5 cm^2^).

The mass of the dried polymer was estimated by using the Sauerbrey equation [54], that relates the mass change per unit area at the electrode surface to the observed change in oscillation frequency of the crystal:

(1) $$\Delta f=\frac{-2f_{0}^{2}}{A\sqrt{\rho_{q}{µ}_{q}}}\Delta m$$

where *f*_0_ is oscillation frequency of the fundamental mode of the EQCM, *A* is crystal area (1.37 cm^2^), $\rho_{q}$ is density of quartz (2.648 g cm^−3^), and μ_q_ is the shear modulus of quartz (2.947 × 10^11^ g cm^−1^ s^−2^). In the case of the device used in this work, the constant may be substituted with the sensitivity factor *C*_f_ (56.6 Hz μg^−1^ cm^2^), resulting in the Equation (2). The value of *C*_f_ was calibrated by the manufacturer. A change in this value is unlikely as deposition of the polymer film was conducted homogeneously and the electrochemically active area covered most of the crystal surface [55].

(2) $$\Delta f=-C_{f}\Delta m$$

As the Sauerbrey equation is only applicable in this form to thin rigid films, i.e., when the product of immobilized film viscosity and density does not change significantly throughout the experiment, we monitored the viscoelastic phenomena via the motional resistance of the crystal to comply with the condition of a thin film [56].

The scan rates for deposition and the number of polymerization cycles and times were adjusted for each polymer to produce approximately 4.2 ± 0.1 μg of the dry polymer on the electrode surface. The electrode with the deposited polymeric film was rinsed with acetonitrile and dried in argon for 30 min. Then the electrode was transferred to the supporting electrolyte solution and electrochemical measurements were performed in potential range 0–1.4 V at different scan rates.

The number of electrons exchanged by each poly-[Cu(Schiff)] unit during redox conversion of the polymer, *n*, was calculated using the following equation [54]:

(3) $$n=\frac{\Delta Q\cdot M}{F\cdot \Delta m}$$

where *Q* is the amount of charge passed in electrochemical reduction of a poly-[Co(Schiff)] film in C; *M* is a molar mass of a polymer fragment in g/mol; *F* is the Faraday constant (96,485 C mol^−1^); *m* is a mass of the poly-[Cu(Schiff)] film determined by QCM, g.

The values of specific capacity, *C_mAh_* in mAh g^−1^, were calculated for the investigated polymers using equation:

(4) $$C_{mAh}=\frac{n\;F}{M_{CuSchiff}*3.6}$$

### 2.3. In Situ Conductance Measurements

For conductivity tests, the polymers were synthesized on an interdigitated platinum electrode (MicruX Technologies, Asturias, Spain) according to the methods mentioned above until the charge of synthesis reached 20 mC. The stability test, combined with in situ conductivity measurement, was carried out by potentiodynamic cycling from (0.0–0.8 V) 0.0 to 1.4 V with scan rate 0.01 V s^−1^ during 5 cycles.

The conductivity-potential profile of the polymers was determined by cyclic voltammetry on an interdigitated electrode (IDE) with a constant potential bias between grids of IDE with a bipotentiostate. As described in [8,57] in more detail, for conductivity measurements, bipotentiostat sets the potential profile of each grid in such a way that the potential difference between working electrode 1 WE(1) grid and working electrode 2 WE(2) grid to be a constant (we used 5, 10, and 20 mV differences). During the potential scanning, the current flowing through the working electrodes, WE(1) and WE(2), involves the faradaic current of the electrochemical process and the leakage ohmic current between grids. Assuming that the value of the Faraday currents is the same for both working electrodes as polymer thickness over the whole IDE electrode is uniform and a low potential bias between two electrodes applied, we can write that:

(5) $$I_{WE\left(1\right)}=I_{F}-I$$

(6) $$I_{WE\left(2\right)}=I_{F}+I$$

(7) $$I=\frac{I_{WE\left(2\right)}-I_{WE\left(1\right)}}{2}\;$$

where $I_{WE\left(1\right)}$ and $I_{WE\left(2\right)}$ are currents passing through the first and second electrodes, *I* is the leakage current between the working electrodes, and $I_{F}$ is the faradaic current.

Finally, conductivity σ and resistance R could be calculated according to the formula:

(8) $$\sigma =\frac{1}{R}=\frac{\Delta I}{2V}$$

where V is the potential difference between the working electrodes and ΔI is the difference between the currents on working electrodes. We checked that leakage currents were following the Ohm law within 5, 10, and 20 mV biases and that CV curve of the polymer on IDE electrode with shorted grids reasonably reassembles CV curve obtained with the (7) at bipotentiostate mode on the same polymer-coated IDE. Double-control results ensure that assumptions of faradaic currents equality for both grids of IDE are safe.

### 2.4. In Situ Spectroelectrochemistry

Studies of the electrochemical properties of the polymers were carried out using modular potentiostat/galvanostat Bio-Logic (BioLogic Science Instruments, Seyssinet-Pariset, France). Polymers were synthesized in a handmade three-electrode cell consisting of a working electrode, a counter electrode (glassy carbon plate S = 2 cm^2^), and a reference electrode. We used an ITO coated glass slide (Sigma Aldrich) as a working electrode. Polymers were synthesized in the form of thin fims on a conducting surface by electropolymerization from a solution of 0.001 mol dm^−3^ [Cu(3-MeOSal-Sal)en] and 0.1 mol dm^−3^ Et_4_NBF_4_ in acetonitrile. Electrochemical polymerization was performed under conditions identical to Section 2.2.

UV-vis absorption spectra of poly-[Cu(3-MeOSal-Sal)en] were registered by a spectrophotometer SF-2000 (Russia). UV-vis spectroscopy of the polymer films deposited on transparent electrodes was carried out in a photometric cell and in situ absorption spectra were registered during potentiostatic polarization measurements. This provided direct information about the polymer structure at any oxidized, reduction, or semi-oxidized state in visible UV or near infrared ranges of light. Film potential was measured against a silver chloride-coated silver wire as the pseudo-reference electrode (the potential of 0 V vs. Ag/AgCl,sat’d NaCl). The spectra were recorded after achieving a stationary state (until current became a constant for approximately 100–150 s).

### 2.5. X-ray Photoelectron Spectroscopy (XPS)

The X-ray Photoelectron Spectroscopy (XPS) of the materials was carried out on the Thermo Fischer Scientific Escalab 250Xi Spectrometer (Thermo Scientific, MA, USA) with nonmonochromatic Al Kα radiation (photon energy 1486.6 eV) at a research park of St. Petersburg State University Centre for Physical Methods of Surface Investigation (Russia). The total energy resolution of the experiment was about 0.2 eV. Spectra of the samples were recorded in the constant pass energy mode at 20 eV using a 650 mm diameter analysis area. Investigations were carried out at room temperature in an ultrahigh vacuum of the order of 10^−9^ mbar.

### 2.6. Scanning Electron Microscopy (SEM)

Studies of the polymers’ morphology were carried out using scanning electron microscopy. Polymers were synthesized on a silicon dioxide piezoelectric crystal sprayed with a layer of platinum in the same mode (see above). The cyclic voltammograms of polymers 1, 2, and 3 were registered in a supporting electrolyte by potential cycling in the range 0 ÷ 1.4 V, 0 ÷ 0.85 V, and 0 ÷ 1.4 V, respectively. Then the samples were washed in pure acetonitrile and dried.

The registration of SEM images was carried out at 45° to the surface of the electrode. The scanning electron microscope (Zeiss Merlin, Jena, Germany) with a 5 kV accelerating voltage was used at a research park of St. Petersburg State University: Interdisciplinary Center for Nanotechnology.

## 3. Results

### 3.1. Electrochemical Behavior of Monomer [Cu(3-MeOSal-Sal)en]

**Figure 1:**
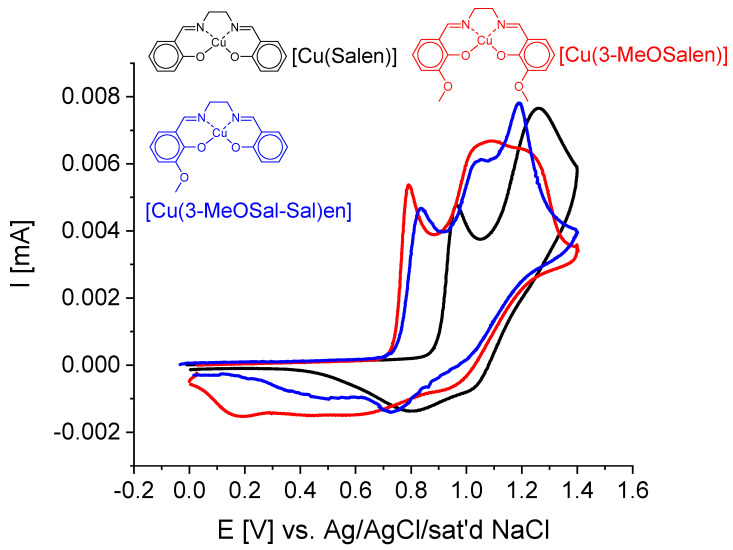
Monomers oxidation at a Pt-electrode (0.02 cm^2^): first scan in 0.1 mol dm^−3^ Et_4_NBF_4_/CH_3_CN and 0.001 mol dm^−3^ [Cu(Schiff)] at 0.05 V s^−1^.

The CV curves of monomeric copper complexes with different Salen ligands-[Cu(Salen)], [Cu(3-MeOSalen)], and [Cu(3-MeOSal-Sal)en]—are shown in Figure 1. Anodic oxidation of [Cu(Salen)] and [Cu(3-MeOSalen)] complexes is well studied in the literature [27,29,43] and has been reperformed here for comparison.

The CV curves of these symmetrical complexes reveal two distinct peaks corresponding to two redox processes. The first process can be attributed to the one-electron oxidation of the monomeric complexes with the phenoxyl radical formation, [Cu(Salen)]^•+^ and [Cu(3-MeOSalen)]^•+^, respectively. The peak potential of the [Cu(3-MeOSalen)] oxidation (0.79 V) is in a less positive region compared to the [Cu(Salen)] complex (0.94 V). The presence of electron-donor substituents in the ligand of the [Cu(3-MeOSalen)] complex increases the electron density in the phenyl rings, thereby facilitating the process of electron transfer [27].

The second oxidation peak corresponds to the second electron transfer with the formation of the biphenoxyl radical species [Cu(Salen)]^••++^ and [Cu(3-MeOSalen)]^••++^, respectively [58]. The second oxidation peak potential of the [Cu(3-MeOSalen)] complex (a broad peak at aprox. 1.2 V, composed of two strongly overlapping peaks) is also located in a less positive region than the potential of the oxidation peak of the [Cu(Salen)] complex (1.3 V) due to the electron-donor effect of methoxy groups of the [Cu(3-MeOSalen)] complex. The coupling of phenoxyl and biphenoxyl radicals through the para-position of the phenyl rings leads to the deposition of a polymer film on the electrode surface [29,45].

For the asymmetric [Cu(3-MeOSal-Sal)en] complex, two main oxidation peaks are observed at 0.83 and 1.19 V, with a shoulder at the potential of 1.05 V (Figure 1). The oxidation onset of the asymmetric monomeric complex [Cu(3-MeOSal-Sal)en] coincides with the oxidation onset of the symmetric monomeric complex [Cu(3-MeOSalen)]. However, the peak potential of the first redox process is shifted by 40 mV to the region of more positive potentials. The next redox process occurs in the same potential range as in the case of the [Cu(3-MeOSalen)] complex (0.9–1.3 V). However, a clear separation of two redox processes occurs at potentials of 1.05 and 1.19 V. Based on the above observations, it can be assumed that the *MeO*-substituted fragment of the asymmetrical molecule is oxidized first and the one-electron oxidation of the monomer leads to the formation of phenoxyl radical [Cu((3-MeOSal)^•+^-(Sal))en]. The second oxidative process involves an additional electron transfer to form a biphenoxyl radical [Cu(3-MeOSal-Sal)en]^••++^.

### 3.2. Polymerization of [Cu(3-MeOSal-Sal)en]

The phenoxyls of asymmetric monomeric complex [Cu(3-MeOSal-Sal)en] can be selectively oxidatively activated at different potentials. At mild potentials, only the methoxylated phenoxyl radical formation occurs, while at higher potentials, dication diradical species are presumable intermediates of the polymerization process. (Scheme 1).

Two approaches are used for the electrochemically induced polymerization of [Cu(3-MeOSal-Sal)en]:

(i) deposition under potentiodynamic control. The films were obtained by scanning the potential in a wide potential range of 0–1.4 V (scan rate 0.05 Vs^−1^). At this type of film deposition, any combination of crosslinkable fragments is possible: MeOPh-MeOPh, Ph-Ph, or MeOPh-Ph, since the applied potential is sufficient to oxidize both phenyl rings. The polymer obtained using this method will have a random structure and is denoted as **polymer 1**.

(ii) deposition under potentiostatic control. The films were obtained by slow deposition at a potential of 0.85 V; that value corresponds to the beginning of the oxidative polymerization process. In this case, the formation of C-C bonds occurs only via methoxy-substituted phenyl ring activation (Scheme 1). The polymer film obtained under potentiostatic control is denoted as **polymer 2**. It is expected to have a uniform and ordered structure and predominantly consist of the dimeric units. NMR analysis of **polymer 2** acid hydrolysis products (Figures S1–S3) revealed a 2.5-to-1 ratio of di-methoxylated biphenyl to mono-methoxylated biphenyl. No unmethoxylated biphenyl was found using NMR. Thus, **polymer 2** has 71.5% MeOPh-MeOPh, 28.5% MeOPh-Ph, and 0% Ph-Ph linkages.

In addition, after the formation of the **polymer 2** film in the monomer solution under potentiostatic control, the film was transferred to a supporting electrolyte solution where it was further oxidized by cyclic potential scan up to 1.4 V. The polymer film obtained by cross-linking of the **polymer 2** is denoted as **polymer 3**.

### 3.3. Electrochemical Behavior Poly-[Cu(3-MeOSal-Sal)en]

**Figure 2:**
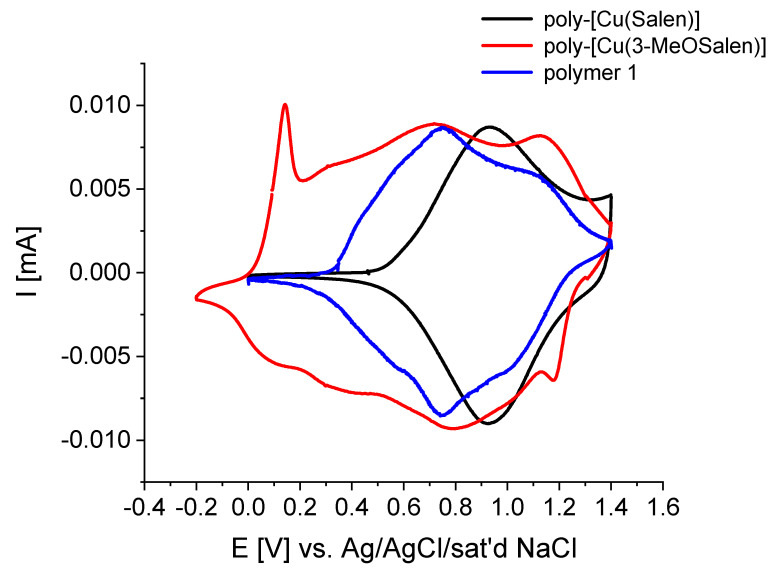
Cyclic voltammograms of the polymers at a Pt-electrode (0.02 cm^2^): stable scan after polymerization in 0.1 mol dm^−3^ Et_4_NBF_4_/CH_3_CN at 0.05 V s^−1^.

CV curves of symmetrical copper polymers and **polymer 1** are shown in Figure 2. All polymers were obtained in the potentiodynamic polymerization mode under the same conditions. One can observe that the introduction of electron-donor methoxy-substituents into the ligand shifts the electroactivity region of the complexes towards less positive potentials. The introduction of one methoxy group into the ligand structure (poly-[Cu(3-MeOSal-Sal)en], **polymer 1**) shifts the oxidation onset of the complex by 0.15 V compared to the poly-[Cu(Salen)] without substituents. The presence of two methoxy groups in the ligand of poly-[Cu(3-MeOSalen)] further shifts the oxidation onset to the less positive potentials region compared to the unsubstituted poly-[Cu(Salen)] (Figure 2) [27].

**Figure 3:**
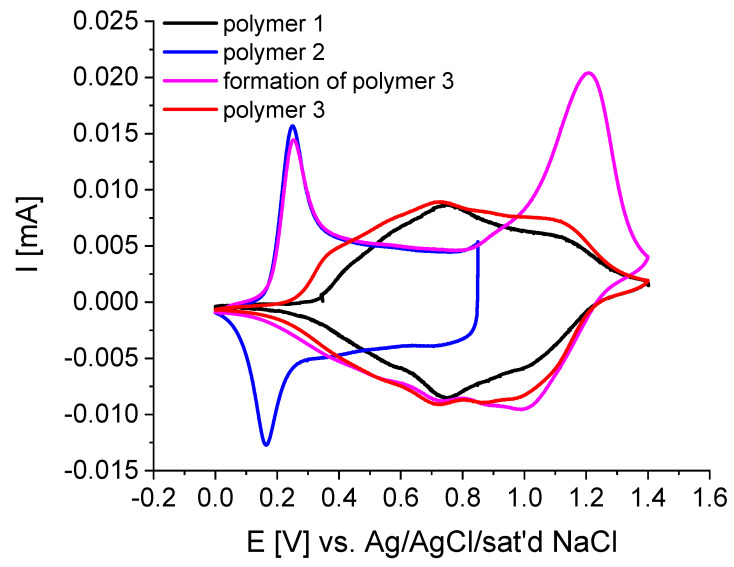
Cyclic voltammograms of a poly-[Cu(3-MeOSal-Sal)en] film (**polymer 2,** black curve) and **polymer 3** (red curve) at a Pt electrode (0.02 cm^2^) in 0.1 mol dm^−3^ Et_4_NBF_4_/CH_3_CN at 0.05 V s^−1^. Additional oxidation of **polymer 2** with the formation of **polymer 3** is marked with a magenta line.

The CV curve of **polymer 2** obtained by the potentiostatic mode has a pair of sharp redox peaks (blue curve, Figure 3). They correspond to the redox processes in the film and are located in the same potentials as the initial stage of oxidation of the polymer of symmetrical [Cu(3-MeOSalen)].

Excursion of **polymer 2** to higher potentials leads to the formation of **polymer 3** (Figure 3, red curve). Oxidation of **polymer 2** causes an additional irreversible peak at a potential of 1.2 V, that is the same with the potential for the formation of biphenoxyl radicals during film formation in the monomer solution (Figure 3, magenta curve). More than 30% of the total oxidation charge is spent on the irreversible oxidation of the unsubstituted phenyl rings of the polymer film. Further cycling of **polymer 3** gives a reversible CV curve that mimics the shape of the CV curve of **polymer 1**, except that **polymer 3** exhibits a wider electroactivity range than **polymer 1**.

We conducted a series of cyclic voltammetry measurements at different scan rates. Figure S5a,b illustrates a thin layer/surface-type regime for charge transport.

To evaluate the electrochemical capacity and gain insights into mass transport during electrochemical doping of the new polymers, they were additionally studied by the EQCM method in a monomer-free electrolyte. Electrochemical oxidation of the polymers is accompanied by an increase in their weight due to injection of charge-compensating ions and solvent molecules into the film; this reduction is accompanied by corresponding species ejection the film (Figure S4).

For all the studied polymers, one linear region was observed on Δ*m*(*Q*) curves (Figure S4). For **polymers 1** and **3**, the same masses of charge carriers of approx. 50 g mol^−1^ was found. This corresponds to the entry of BF_4_¯ anions into the film with the simultaneous ejection of solvent molecules CH_3_CN (Table 1). Thus, during the oxidation/reduction of polymers in the full range of potentials, there are no significant differences in the charge-transferring species. When **polymer 2** is oxidized in the range from 0 V to 0.85 V, anions BF_4_¯ (molar mass 87 g mol^−1^) inject into the film without the ejection of solvent molecules.

We combined the results of the electrochemical studies with the data on the polymer mass obtained by QCM for dried dedoped films to determine the number of electrons per a monomer unit exchanged by investigated poly-[Cu(3-MeOSal-Sal)en] films during their oxidation/reduction (n). Calculated values of n are shown in Table 1. As can be seen, all poly-[Cu(3-MeOSal-Sal)en] films investigated in the present study exchange more than one electron per monomer unit in the oxidation/reduction processes. **Polymer 3** demonstrates the highest number of electrons reversibly exchanged per monomeric unit of the polymer.

The multielectronic nature of the oxidation/reduction processes in all polymers makes them viable candidates for energy storage applications. Table 1 lists the values of specific capacity (C_mAh_ in mAh g^−1^) calculated for the studied polymers using Equation (4).

The calculated capacity for **polymer 3** is 25% higher than for **polymer 1**. The specific capacity for **polymer 3** is on par with the numbers reported for conductive polymers for use in electrochemical energy storage [11,59].

### 3.4. In Situ Conductance Studies of Polymers Films

**Figure 4:**
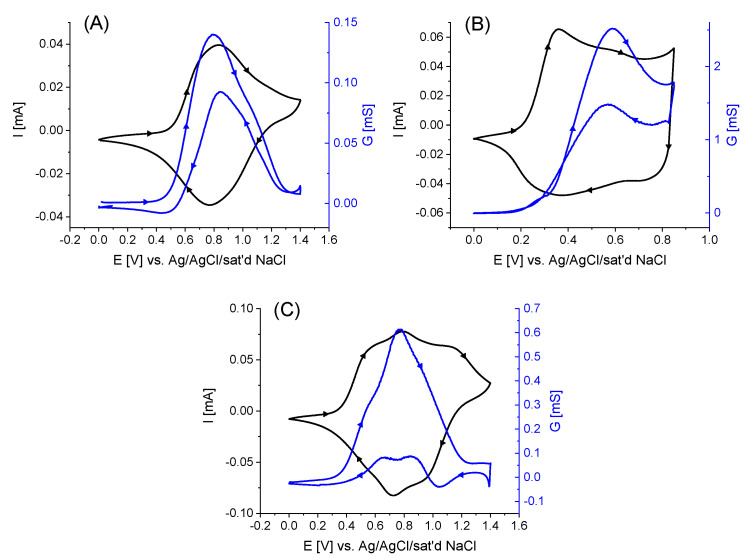
The conductivity of polymers measured using interdigitated electrode arrays in in 0.1 mol dm^−3^ Et_4_NBF_4_/CH_3_CN recorded during CV with 5 mV s^−1^: **polymer 1** (**A**), **polymer 2** (**B**), **polymer 3** (**C**).

The dependence of poly-[Cu(3-MeOSal-Sal)en] polymer conductivity versus potential was studied using IDE electrodes. Figure 4 shows the conductivity of the polymer complexes in line with their CV curves.

A linear increase in the conductivity of **polymers 1** and **3** is observed at the onset of film oxidation. The maximum of conductivity coincides with the maximum of the main oxidation peak of the voltammograms. In this state, the polymer is in half of the maximum doping state. Further increase of the potential leads to a decrease in conductivity. The conductivity value approaches zero when the polymer is fully oxidized. In this state, mobile charge carriers are almost completely absent. The reverse scanning of the potential is accompanied by an increase in conductivity, with the maximum being reached at the potential of the reverse peak. With a further decrease in the potential, the conductivity falls to zero; since the film is in a completely reduced state, there are no mobile charge carriers.

The conductivity profiles of **polymers 1** and **3** show several peaks that pointed to several types of charge carriers. The conductivity profile of **polymer 1** contains one weakly resolved conductivity maximum at 0.6 V and a pronounced maximum at 0.8 V. The conductivity profile of **polymer 3** contains at least two peaks located at the same potentials as the first and second CV waves.

The conductivity profile of **polymer 2** shows one peak at a potential corresponding to a weakly resolved voltammogram peak at 0.6 V. The conductivity of the film begins to grow from the beginning of its oxidation. However, the maximum conductivity was attained at more positive potentials than the potential of the main peak (0.35 V). **Polymer 3** exhibits a higher conductivity than **polymer 1** and a lower conductivity than **polymer 2**.

### 3.5. In Situ UV-vis Spectroelectrochemistry

To probe the nature of species induced in polymers by electrochemical oxidation, spectroelectrochemical analysis of the polymers at different doping levels was performed. Figure S6 shows the UV-vis spectra of poly-[Cu(3-MeOSal-Sal)en] films on an ITO-coated glass electrode acquired during redox switching. Figure S7 shows the characteristic absorption spectra in a differential form, obtained by subtracting the spectrum of the reduced polymer (at the potential 0 V) from the spectra recorded at all other potentials. The spectra of the polymeric symmetrical complexes poly-[Cu(Salen)] and poly-[Cu(3-MeOSalen)] was measured under the same experimental conditions and will be used to interpret the bands in the UV-vis spectrum of the polymeric unsymmetrical complex poly-[Cu(3-MeOSal-Sal)en].

**Figure 5:**
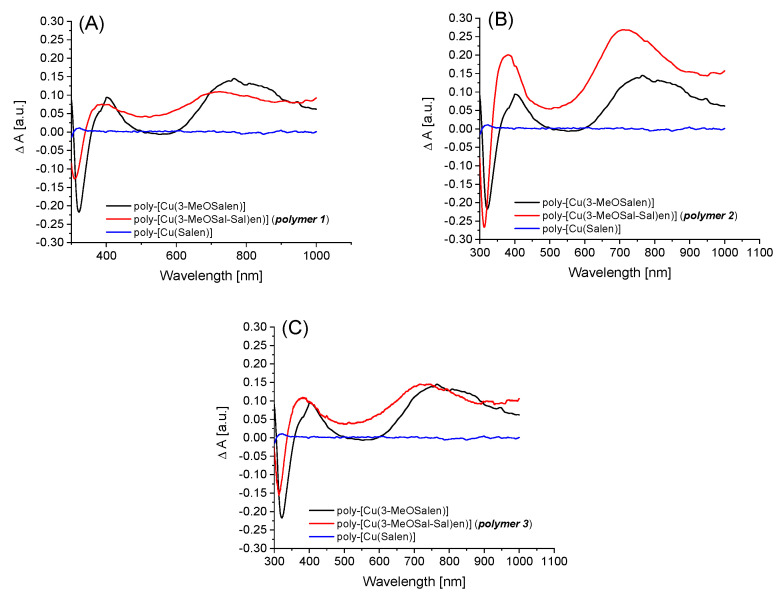
Comparison of UV-vis spectra of poly-[Cu(3-MeOSal-Sal)en] (**polymer 1** (**A**), **polymer 2** (**B**), **polymer 3** (**C**)), with poly-[Cu(Salen)] and poly-[Cu(3-MeOSalen)] films at an ITO-coated glass electrode acquired during redox switching in 0.1 mol dm^−3^ Et_4_NBF_4_/CH_3_CN at the low doping level (E = 0.6 V).

UV-vis spectra of all poly-[Cu(Schiff)] polymers have an absorption band at 313 nm ascribed to π-π* intraligand charge transfer transitions. With the increase in the doping level of polymer (potential increase), the intensity of the band decreases. At a low doping level, the spectrum of the symmetrically substituted poly-[Cu(3-MeOSalen)] contains the intense bands at 400 nm and 790 nm. These two bands are attributed to the formation of Cu(ΙΙ)—phenoxyl cation radicals in the film [33,60,61], localized on the ligand (Figure 5). At the same time, no changes occur in the spectrum of the unsubstituted poly-[Cu(Salen)] polymer at potentials up to 0.6 V; the spectrum coincides with the spectrum of the reduced form. Consequently, the transitions at 380 nm and 720 nm in the spectrum of asymmetric poly-[Cu(3-MeOSal-Sal)en] (**Polymers 1, 2,** and **3**) upon initial oxidation are related to the formation of cation radicals localized on the methoxy-substituted phenyl rings. The intensity of these bands is highest for **polymer 2** among all three polymers. A hypsochromic shift of the bands is observed for the poly-[Cu(3-MeOSal-Sal)en] at a low doping level compared to the poly-[Cu(3-MeOSalen)].

**Figure 6:**
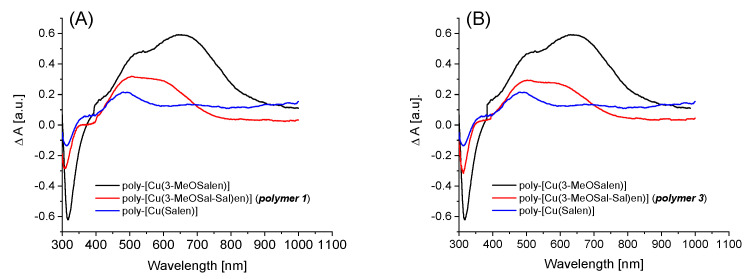
Comparison of UV-vis spectra of poly-[Cu(3-MeOSal-Sal)en] (**polymer 1** (**A**), **polymer 3** (**B**)), with poly-[Cu(Salen)] and poly-[Cu(3-MeOSalen)] films at an ITO-coated glass electrode acquired during redox switching in 0.1 mol dm^−3^ Et_4_NBF_4_/CH_3_CN at the high doping level (E = 1.4 V).

With an increase in the electrode potential, the polymers poly-[Cu(Salen)] and poly-[Cu(3-MeOSalen)] exhibit an increase in the bands at 490 nm and 690 nm and 530 nm and 655 nm, respectively (Figure 6). These bands are attributed to the formation of dications in the film [28,60,61,62]. **Polymers 1** and **3** contain a pair of overlapping bands in the same wavelength region (502 nm and 610 nm). These bands are also attributed to the formation of dications in the polymer films. The decrease in intensity of the band at 380 nm in the completely doped polymeric film indicates the conversion of cation radicals into dications. However, the intensity of the bands at low doping levels for **polymer 3** is somewhat higher than for **polymer 1**. This may indicate a higher concentration of cation radicals in the film.

Some works devoted to elucidation of the localization of positive charge in oxidized protected [Cu(Salen)] found evidence of Cu(ΙΙΙ)—phenolates formation [32,33,35,63]. The band corresponding to the LMCT transition in Cu(ΙΙΙ)-phenolate species is located in the region of 550 nm [32,35]. In the spectra of asymmetric polymer complexes poly-[Cu(3-MeOSal-Sal)en]^•+/2+^, the absorption is observed in this wavelength range without any defined band. This absorption is likely due to the superposition of broad neighboring bands; as such, it is not possible to unambiguously interpret this region of wavelengths.

The results of spectroelectrochemical studies revealed that the redox processes in poly-[Cu(3-MeOSal-Sal)en] films are complex and that participation of a metal center in the redox processes in the polymers could not be excluded.

### 3.6. XPS

The XPS method can be used to prove the participation of the copper of the polymer complexes in redox processes.

For **polymer 1**, XPS data were used to estimate the oxidation state of the central copper atom (Figure S8). A comparative analysis of the binding energy of 2p Cu electrons for the solid monomer and the polymer in the oxidized state showed a difference towards higher energies for the polymer compound. A shift of 2.3 eV in the binding energies of Cu 2p^3/2^ (from 932.3 to 934.6 eV) and Cu 2p^1/2^ (from 952.0 to 954.4 eV) is consistent with the change in the oxidation state of Cu(ΙΙ) to Cu(ΙΙΙ) [32,33,35]. The broadened satellite peak in the form of a weakly resolved triplet, the shape of which is determined by the multiplet splitting of the 3D states of copper, is likely due to the tetragonal distortion of the structure of the complex. Thus the copper atom acts as a separate redox center in the poly-[Cu(3-MeOSal-Sal)en]. A more detailed electronic structure of 3D orbitals of the copper atom and spin-allowed transitions is shown in the Scheme S1.

### 3.7. SEM Studies of Polymers

**Figure 7:**
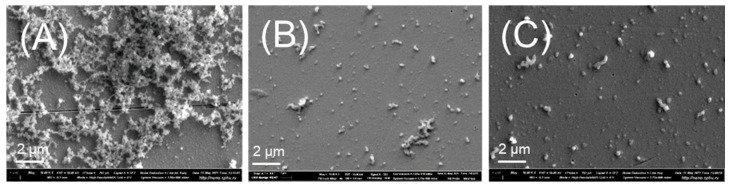
Scanning electron micrographs of poly-[Cu(3-MeOSal-Sal)en] films deposited on a Pt-electrode: **polymer 1** (**A**), **polymer 2** (**B**), **polymer 3** (**C**).

Figure 7 shows SEM images of the polymers. For **polymer 1**, formed in the full range of potentials, there are more globular structures on the surface. **Polymer 1** has a relatively flexible organic framework and forms a globular material with pore inclusions (Figure 7a).

**Polymer 2** has a uniform surface with very low visible porosity (Figure 7b). It can be assumed that the polymer consists of tight stacks of rigid monomers and has a homogeneous structure. One-level relief is primarily observed.

**Polymer 3** also has a homogeneous structure (Figure 7). However, a small number of globules is observed on the surface, as in the case of **polymer 1**. More SEM-images can be seen in Figure S9.

## 4. Discussion

An asymmetrical monomer design provides new possibilities for fine-tuning the properties of conductive electroactive polymers composed of these building blocks. The asymmetrical copper complex poly-[Cu(3-MeOSal-Sal)en] structure has two fragments with different electron saturation: a fragment of electron-unsaturated phenyl ring and a fragment of a methoxy-substituted electron-saturated phenyl ring. By varying the electropolymerization potential, polymer films with different combinations of crosslinked fragments have been obtained.

Under potentiodynamic polymerization with a wide potentials range, **polymer 1** with randomly crosslinked phenyl rings was obtained. **Polymer 2** was obtained under potentiostatic deposition at a low potential. Under these conditions, only activation of the methoxy-substituted phenyl sites is possible. This polymerization pathway is confirmed by the NMR analysis of **polymer 2** hydrolysis products. Moreover, the presence of MeOPh-Ph biphenyls in **polymer 2**’s structure allows us to propose the significance of the radical-substrate coupling mechanism of C-C bond formation for this system under the conditions studied. Asymmetrical ligand design allows the C-C coupling radical-radical dimerization and radical-substrate mechanisms to be distinguished in a way that, to the best of our knowledge, has never been conducted for these complexes. More precise control over oxidative coupling conditions may favor dimer formation and further improve structural control under polymerization pathway.

**Polymer 3** is characterized by a more regular alternation of monomeric fragments when compared with **polymer 1**, since it was obtained by further crosslinking of uncoupled unsubstituted phenyl rings of **polymer 2**.

Oxidation of **polymer 2** is accompanied by the formation of poly-[Cu(3-MeOSal-Sal)en]^•+^ cation radicals with a charge localized in the methoxy-substituted phenyl ring, as evidenced by the UV-spectrum data (the growth of the bands at 380 nm and 720 nm). The concentration of cation radicals, as well as the conductivity value, are the highest among all the studied polymers, indicating a high degree of structural order. It is known that the electrical conductivity of metallopolymers decreases with increased distance between polymer chains [7]. Interchain interactions play an important role in the processes of charge transport in polymeric metal complexes. When two polymer chains interact, a weak π-bond is formed between the aromatic rings of the monomeric fragments. The formation of π-aggregates is facilitated by the segmentation of the polymer structure, i.e., the presence of fragments oxidized at different potentials [64]. The presence of an efficient system of interchain interactions provides bulk conductivity and is a necessary step for achieving high values of specific conductivity. The structure of **polymer 2** provides the most efficient charge transport via π-stacking among all three structures studied.

The structure of **polymer 2** tends to a long-range pathway of charge transport only via π-stacking of methoxy-substituted biphenyl moieties. Each polymer fragment has one electroactive center at potential range of reversible redox processes. However, the number of electrons exchanged by a polymer fragment in redox processes exceeds one.

Poly-[Cu(Schiff)] polymer complexes in the uncharged state are paramagnetic due to Cu(ΙΙ) ions, that are in the low-spin 3*d*^9^ conformation (a detailed discussion of orbitals is given in SI). The one-electron oxidation of polymers results in the formation of cation radicals located on the ligand. In situ UV-vis spectra show the characteristic features of the phenoxyl radical. Since there is an orthogonality between the metal $3d_{x^{2}-y^{2}}$ orbital and the phenolate-based orbital, the primary oxidation can lead to the transformation of monomer fragments Cu(ΙΙ)—phenolate into Cu(ΙΙ)—phenoxyl radicals. However, the formation of diamagnetic 3*d*^8^ Cu(ΙΙΙ)-phenolate particles in poly-[Cu(Salen)] in addition to Cu(ΙΙ)-phenoxy radicals cannot be completely ruled out, since an equilibrium can exist in the one-electron oxidation of the polymer (Cu(ΙΙ) -phenoxyl radicals ↔ Cu(III)-phenolates). Both types of particle have very similar spectroscopic characteristics in the UV-vis region.

The existence of Cu(III) in the **polymer 2** structure of the oxidized film is indirectly indicated by the presence of a shoulder at a potential of 0.6 V in CV (Figure 4) and the number of electrons exchanged by the polymer fragment in redox processes is greater than 1. The nature of the second peak in the voltammogram can be attributed to the formation Cu(III)-phenolates, since the equilibrium takes place during one-electron oxidation of the film (phenoxyl radicals ↔ Cu(III)-phenolates).

The properties of **polymers 1** and **3** are similar, despite **polymer 1** being obtained by rapid deposition in the full potential range. **Polymer 3** initially had the highly ordered structure of **polymer 2** and then was further oxidatively cross-linked. According to the spectra, both polymers have the same set of bands and, consequently, the same nature of charge carriers. Oxidation of **polymers 1** and **3** results in the formation of cation radicals that are converted into dications with increasing potential. However, for **polymer 3**, the intensity of the bands corresponding to the formation of cation radicals is higher than for **polymer 1**, and hence the concentration of cation radicals in **polymer 3** is higher. This explains the higher specific capacity of **polymer 3** when compared to **polymer 1**, since a greater number of cation radicals can be converted into dications.

The number of electrons exchanged by a polymer fragment during oxidation is highest in **polymer 3**. Each fragment of the poly-[Cu(3-MeOSal-Sal)en] has two types of potential redox centers: biphenyl rings and a metal center. During the oxidation of biphenyl fragments, the maximum number exchanged electrons is two. However, the number of electrons per polymer fragment for **polymer 3** exceeds 2 (2.3 electrons), which indicates the participation of copper atom in redox processes, as in the case of **polymer 2**.

**Polymer 3** shows a higher conductivity, a larger number of electrons exchanged in redox processes, and has a wider area of electrical activity compared to **polymer 1**. All this indicates a more uniform and ordered structure of **polymer 3**. **Polymer 3** initially had the highly ordered structure of **polymer 2**, consisting of methoxy-biphenyls organized in π-stacks. As a result of further oxidation, the π-stack structures were partially destroyed through simultaneous cross-linking of the Sal-Sal fragments. The film no longer has the highly ordered structure of **polymer 2** but is significantly more ordered than **polymer 1**, which was obtained by rapid deposition and contains random combinations of fragments. Rapid deposition leads to polymer films with a disordered structure, numerous defects, and frequent chain terminations.

Differences in the structure of the films are also noticeable at the macrolevel. According to SEM photographs, films of **polymers 2** and **3** have a dense and uniform structure with a small amount of small globular formations on the surface. **Polymer 1** has an uneven, porous structure; many large loose structures are present on the film surface.

## 5. Conclusions

By varying the deposition conditions and the composition of the ligand environment of monomeric metal complexes with an asymmetric design, polymer films with desired properties can be obtained. The choice of potential and rate of deposition of unsymmetrical metal complexes poly-[Cu(3-MeOSal-Sal)en] enables control of the coupling site of an asymmetrical monomer. At a low potential, polymer films were obtained with a highly ordered stack structure and high conductivity. Further oxidation leads to a partial disruption of the order and the simultaneous formation of chain structures. A stepwise formation of a polymer film, in which methoxy fragments are cross-linked at the first step, and then unsubstituted fragments crosslinked at the second one, affords significantly improved polymer electrochemical characteristics, achieving more complete oxidation of the film and an increase in its specific capacity by 25%. With this model system, we have shown that asymmetrical monomer design may be successfully implemented to fine-tune polymerization site, conductivity, and capacitive properties for [M(Salen)]-type polymers. Further investigations in electrocatalytic and sensory systems as well as an understanding of the advantages of this approach in polymeric systems with other metal centers are promising and desirable.

## Figures and Tables

**Table 1:** Molar mass of charge-transferring species participating in the polymer oxidation/reduction process and the number of electrons per monomer unit exchanged during redox processes in the polymer films found by the EQCM.

	Poly-[Cu(3-MeOSal-Sal)en]		
	Polymer 1	Polymer 2	Polymer 3
Potential range, V	0 ÷ 1.40	0 ÷ 0.85	0 ÷ 1.40
Molar mass of charge-transferring species, g mol^−1^	52 ± 4	87 ± 4	49 ± 4
Number of electrons per a monomer unit exchanged	1.7 ± 0.1	1.2 ± 0.1	2.3 ± 0.1
Specific capacity, C_mAh_/mAh g^−1^	136	93	170

## Data Availability

The data presented in this study are available on request from the corresponding author.

## Supplementary Materials

The following supporting information can be downloaded at: https://www.mdpi.com/article/10.3390/polym15051127/s1, Figure S1: Major and minor structures of hydrolysis products; Figure S2: ^1^H NMR spectrum of mixture of 4,4′-dihydroxy-5,5′-dimethoxybiphenyl-3,3′-dicarbaldehyde (major) and 4,4′-dihydroxy-5-methoxybiphenyl-3,3′-dicarbaldehyde (minor) in DMSO-*d*_6_; Figure S3: ^1^H{^13^C} NMR spectrum of mixture of 4,4′-dihydroxy-5,5′-dimethoxybiphenyl-3,3′-dicarbaldehyde (major) and 4,4′-dihydroxy-5-methoxybiphenyl-3,3′-dicarbaldehyde (minor) in DMSO-*d*_6_; Figure S4: Cyclic voltammogram of a poly-[Cu(3-MeOSal-Sal)en] film (at a Pt-coated quartz crystal electrode (1.37 cm^2^) in 0.1 mol dm^−3^ Et_4_NBF_4_/CH_3_CN at 0.05 V s^−1^ and a corresponding electrode mass variation with potential for: (a) **polymer 1**, (c) **polymer 2**, (e) **polymer 3**; a mass—charge plot at 0.05 V s^−1^ for this polymers (b, d, e); Table S1: Molar mass of charge-transferring species participating in the polymer oxidation/reduction process, specific capacity, apparent rate constant and the number of electrons per monomer unit exchanged during redox processes in the polymer films; Figure S5: Cyclic voltammogram of a poly-[Cu(3-MeOSal-Sal)en] film at a Pt-coated quartz crystal electrode (1.37 cm^2^) in 0.1 mol dm^−3^ Et_4_NBF_4_/CH_3_CN recorded at different scan rates: **polymer 1** (a), **polymer 3** (b); dependencies of log (I_p_^a^) and log (I_p_^c^) on log v_s_. for polymers: **polymer 1** (c), **polymer 3** (d); Figure S6: *UV-vis* spectra of a poly-[Cu(3-MeOSal-Sal)en] film at an ITO-coated glass electrode acquired during redox switching in 0.1 mol dm^−3^ Et_4_NBF_4_/CH_3_CN: **polymers 1** (a), **polymers 3** (b), **polymers 2** (c); Figure S7: UV-vis spectra of a poly-[Cu(3-MeOSal-Sal)en] film at an ITO-coated glass electrode acquired during redox switching in 0.1 mol dm^−3^ Et_4_NBF_4_/CH_3_CN: **polymers 1** (a), **polymers 3** (b), **polymers 2** (c), referenced to the spectrum of the polymer at −0.3 V; Figure S8: Cu2p XPS spectra of the polymer films; **Scheme S1.** Schematic representation HOMO-LUMO energy gap; Figure S9: Scanning electron micrographs of poly-[Cu(3-MeOSal-Sal)en] films deposited on a Pt electrode: **polymer 1** (a), **polymer 2** (b), **polymer 3** (c). References [32,33,35,65,66,67,68] are cited in the supplementary materials.
