# Supplementary material for: Asymmetric Monomer Design Enables Structural Control of M(Salen)-Type Polymers
Source: Polymers (Basel). 2023 Feb 23;15(5):1127. doi: 10.3390/polym15051127 (PMC10007425; doi:10.3390/polym15051127)
Supplement: Supplementary file 1 [file polymers-15-01127-s001.zip › polymers-1993533-supplementary.pdf]

Supplementary Materials

Asymmetric Monomer Design Enables Structural Control of M(Salen)-Type Polymers

Maria Novozhilova [1], Julia Polozhentseva [1] and Mikhail Karushev [1,2,*]

[1] Ioffe Physical-Technical Institute of the Russian Academy of Sciences (Ioffe Institute), 26 Polytekhnicheskaya str., 194021 St. Petersburg, Russia
[2] Independent Researcher, Astana 020000, Kazakhstan
* Correspondence: mkarushev@gmail.com

Polymer 2 structure analysis was performed in accordance with the methodology presented in the [1]. 100 mg of polymer 2 have been peeled of a 100 cm^2 glassy carbon electrode and stirred in 20 ml of 20% aqueous sulfuric acid for 40 hours at room temperature. Organic hydrolysis products were extracted 4 times with dichloroethane. Then extract was vacuum-dried, and the resulting products (35 mg) were collected and dissolved in d_6-DMSO for NMR identification.

^1H, $^{13}C\{^1H\}$ NMR spectra, experiments were acquired on a Jeol ECX400A spectrometer (400 MHz for ^1H nuclei and 100 MHz for ^{13}C nuclei) in DMSO-d_6. The residual signals of the solvent (DMSO-d_6: 2.50 ppm for ^1H nuclei and 39.6 ppm for ^{13}C nuclei) were used as internal standard.

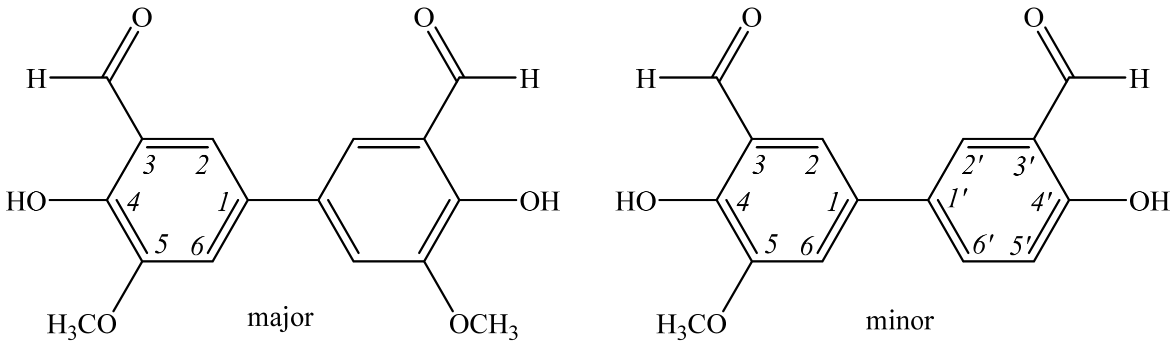

Figure S1. Major and minor structures of hydrolysis products.

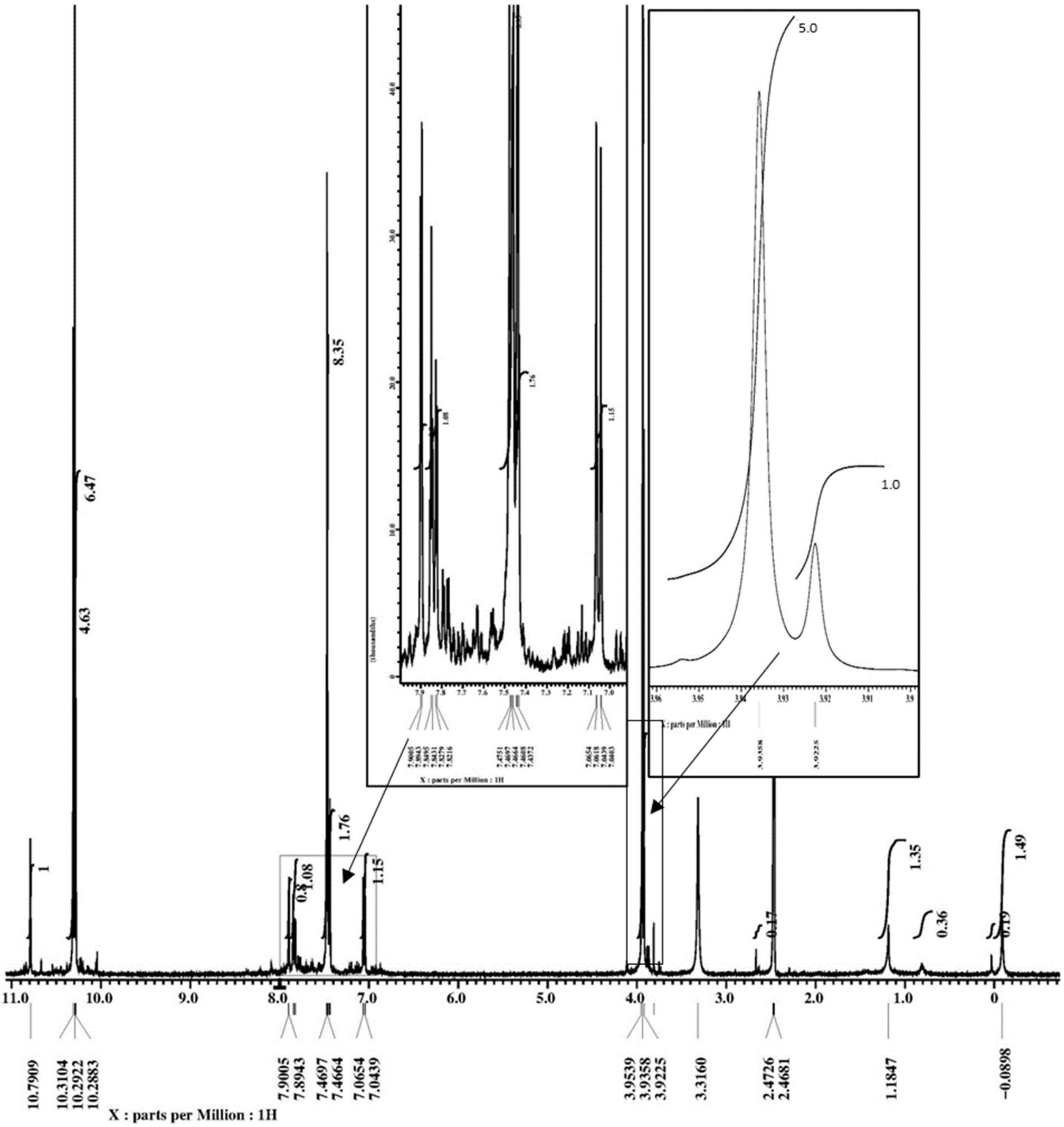

Figure S2. ^{1}H NMR spectrum of mixture of 4,4'-dihydroxy-5,5'-dimethoxybiphenyl-3,3'-dicarbaldehyde (major) and 4,4'-dihydroxy-5-methoxybiphenyl-3,3'-dicarbaldehyde (minor) in DMSO-d_6.

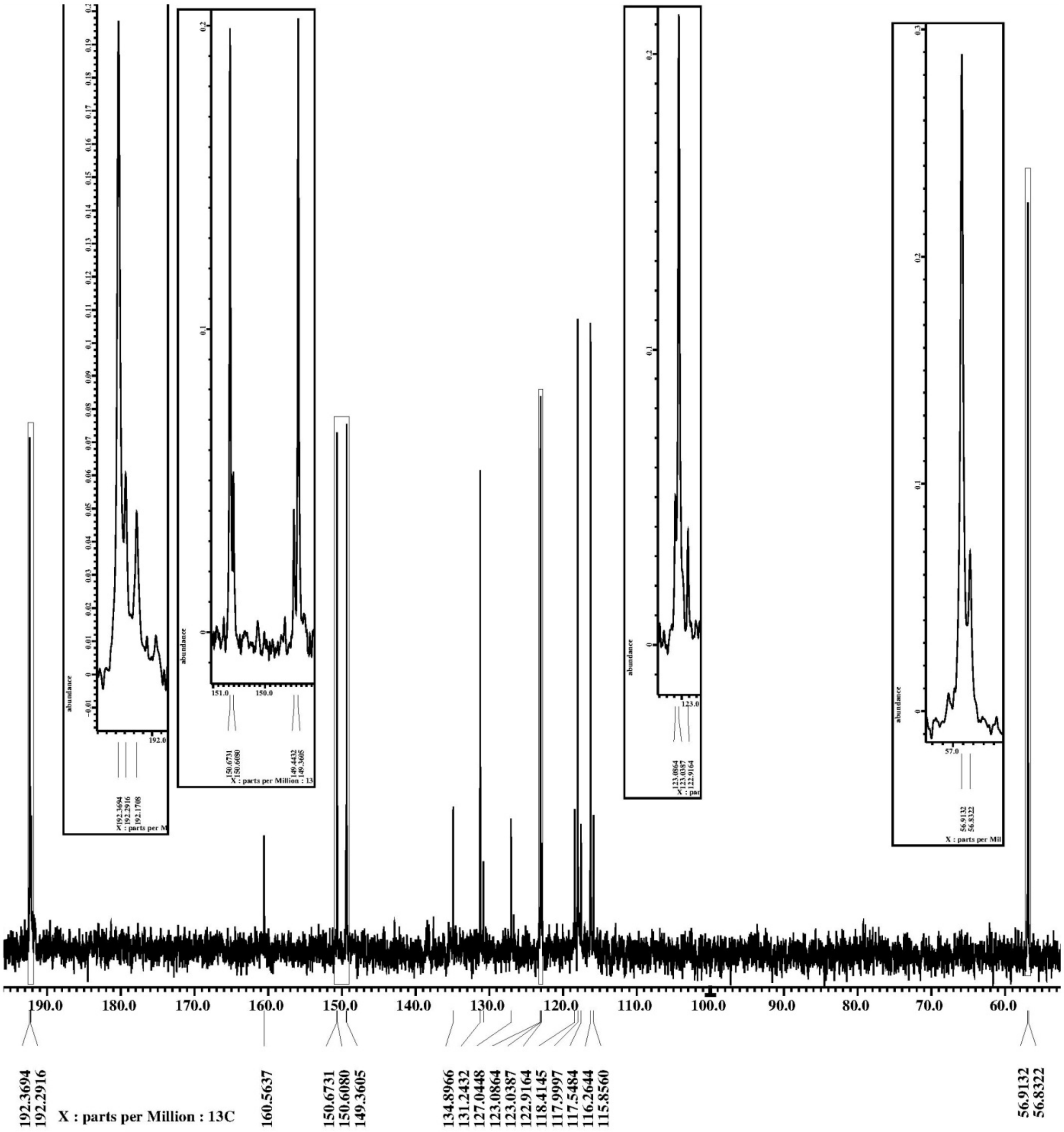

Figure S3. $^1H\{^{13}C\}$ NMR spectrum of mixture of 4,4'-dihydroxy-5,5'-dimethoxybiphenyl-3,3'-dicarbaldehyde (major) and 4,4'-dihydroxy-5-methoxybiphenyl-3,3'-dicarbaldehyde (minor) in DMSO-d_6.

The ratio **1** to **2** is approximately 2.5: 1 and was obtained by integrating the peak corresponding to the signal of the -OCH_3 group(s).

1: ^{1}H NMR: 3.94 (3H, s, OCH_3), 7.46 (1H, d, C^2H or C^6H, ^4J 2.2 Hz), 7.47 (1H, d, C^2H or C^6H, ^4J 2.2 Hz), 10.29 (1H, s, CH=O), 10.31 (1H, s, OH).

$^{13}C\{^1H\}$ NMR: 56.91 (OCH_3), 116.26 (CH Ar), 118.00 (CH Ar), 123.04 (C^3), 131.24 (C^1), 149.36 (C^5), 150.67 (C^4), 192.37 (CH=O).

2: ^{1}H NMR: 3.92 (3H, s, OCH_3), 7.05 (1H, d, $C^{5'}$H, ^3J 8.6 Hz), 7.43 (1H, d, C^2H or C^6H, ^4J 2.2 Hz), 7.44 (1H, d, C^2H or C^6H, ^4J 2.2 Hz), 7.84 (1H, dd, $C^{6'}$H, ^3J 8.6 Hz, ^4J 2.5 Hz), 7.90 (1H, d, $C^{2'}$H, ^4J 2.5 Hz), 10.28 (1H, s, CH=O), 10.29 (1H, s, CH=O), 10.30 (1H, s, C^4-OH), 10.79 (1H, s, $C^{4'}$-OH).

$^{13}C\{^1H\}$ NMR: 56.83 (OCH_3), 115.86, 117.55 ($C^{2,6}$), 118.41 ($C^{5'}$), 122.92, 123.09 ($C^{3,3'}$), 127.04 ($C^{2'}$), 130.79 (C Ar), 134.90 ($C^{6'}$), 149.44 (C^5), 150.61 (C^4), 160.56 ($C^{4'}$), 192.17, 192.29 (2CH=O).

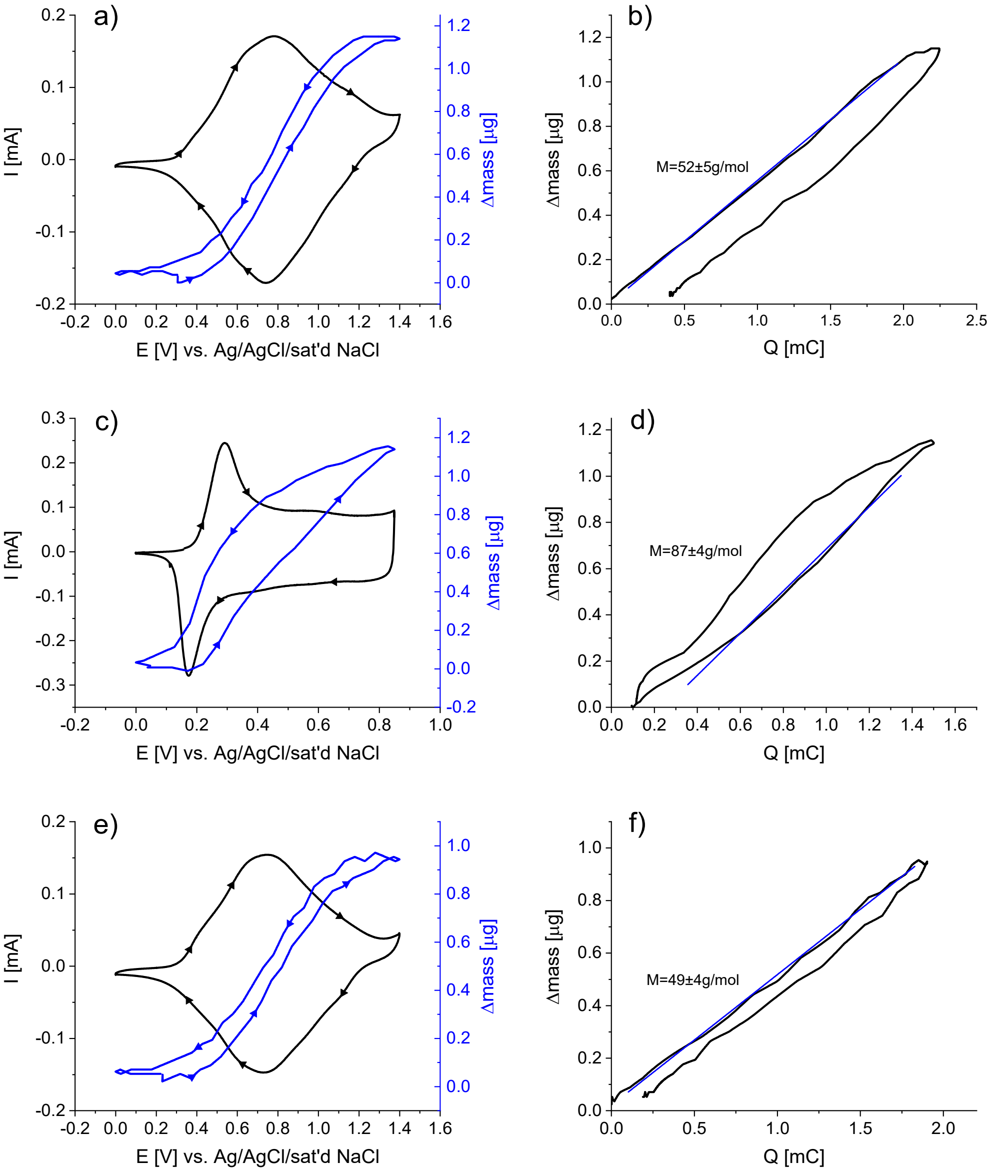

Figure S4. Cyclic voltammogram of a poly-[Cu(3-MeOSal-Sal)en] film (at a Pt-coated quartz crystal electrode (1.37 cm^2) in 0.1 mol dm^{-3} Et_4NBF_4/CH_3CN at 0.05 V s^{-1} and a corresponding electrode mass variation with potential for: (a) **polymer 1**, (c) **polymer 2**, (e) **polymer 3**; a mass–charge plot at 0.05 V s^{-1} for this polymers (b, d, e).

Table S1. Molar mass of charge-transferring species participating in the polymer oxidation/reduction process, specific capacity, apparent rate constant and the number of electrons per monomer unit exchanged during redox processes in the polymer films.

	**Poly-[Cu(Salen)]**	**Poly-[Cu(3-MeOSalen)]**	
Potential range, *V*	**0 ÷ 1.40**	**0 ÷ 0.80**	**0.80 ÷ 1.40**
Molar mass of charge-transferring species, *g mol*$^{-1}$	51±4	45±4	97±4
Number of electrons per a monomer unit	1.0±0.1	1.8±0.1	
Specific capacity, C_{mAh}/*mAh g*$^{-1}$	80	125	
Apparent rate constant, $k_{c,a}^0/s^{-1}$	1.1, 0.9	0.9, 1.1	

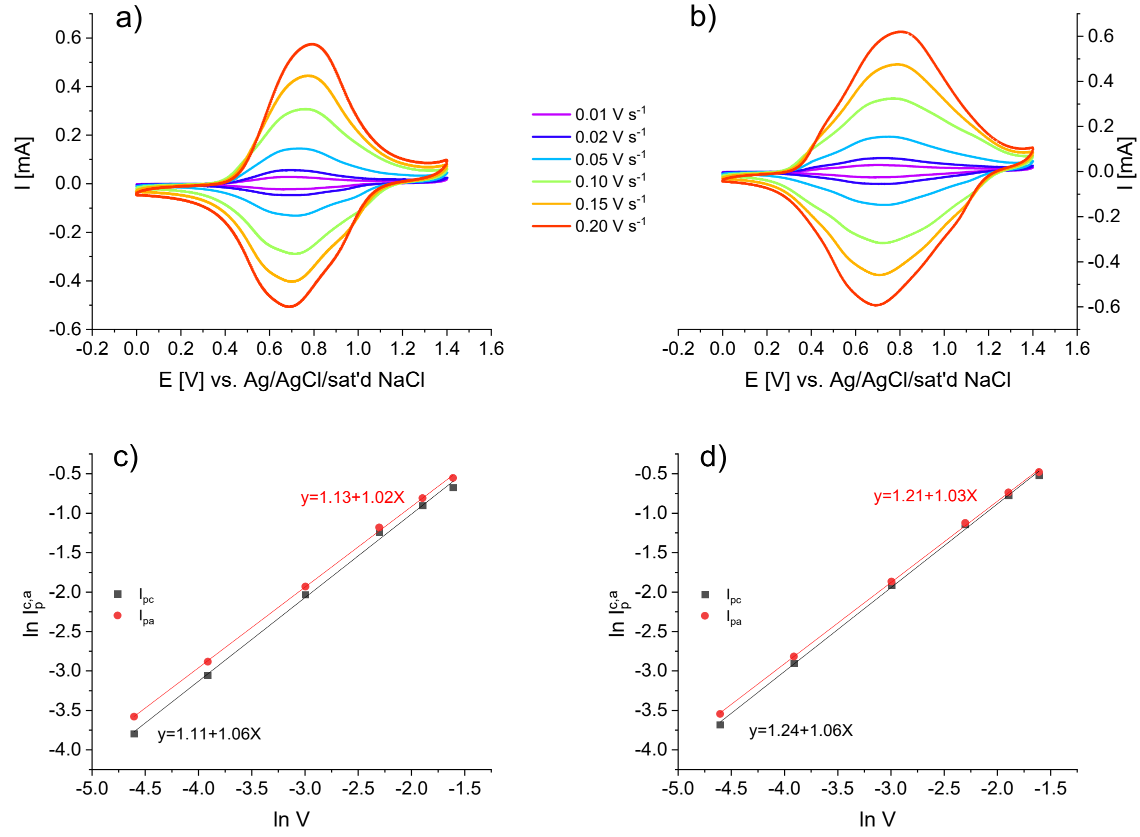

Figure S5. Cyclic voltammogram of a poly-[Cu(3-MeOSal-Sal)en] film at a Pt-coated quartz crystal electrode (1.37 cm^2) in 0.1 mol dm^{-3} Et_4NBF_4/CH_3CN recorded at different scan rates: **polymer 1** (a), **polymer 3** (b); dependencies of log (I_p^a) and log (I_p^c) on log v_s. for polymers: **polymer 1** (c), **polymer 3** (d).

We conducted a series of cyclic voltammetry measurements at different scan rates. Fig.S2 (a, b) illustrates CV curves for scan rates in the range 0.01-0.2 Vs^{-1}.

Testing at different potential sweep rates: peak currents proportional to the potential sweep rate in consistence with the absence of the diffusion limitations for charge transport (fig. S2). For thin film of poly-[Cu(3-MeOSal-Sal)en] and for all scan rates a linear dependence of anodic and cathodic peak current on the scan rate is observed (*log i* versus *log v* plots have slopes close to unity; Fig. S2 (c, d)), which indicates a thin layer/surface-type regime for charge transport. The reaction is limited only by electron transfer.

The apparent rate constants electrode materials were calculated from the dependence of the peak potential on logarithm of the potential sweep rate using standard method [2,3]. The rate constants for poly-[Cu(Salens')] have close values to the rate constant and poly-[Ni(Salens')], showing that the electron transfer proceeds by the same mechanism. Thus, poly-[Cu(Salens')] exhibit fast charge transport kinetics in the fully oxidized state.

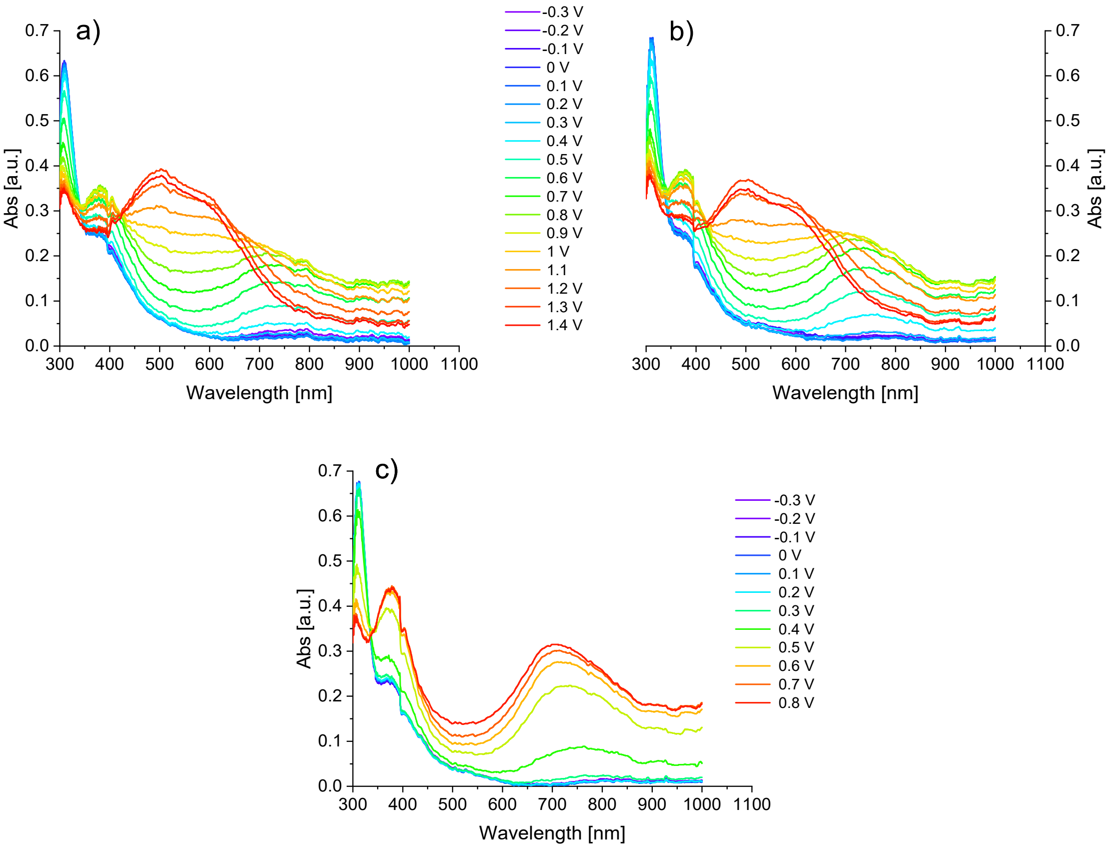

Figure S6. *UV-vis* spectra of a poly-[Cu(3-MeOSal-Sal)en] film at an ITO-coated glass electrode acquired during redox switching in 0.1 mol dm^{-3} Et_4NBF_4/CH_3CN: **polymers 1** (a), **polymers 3** (b), **polymers 2** (c).

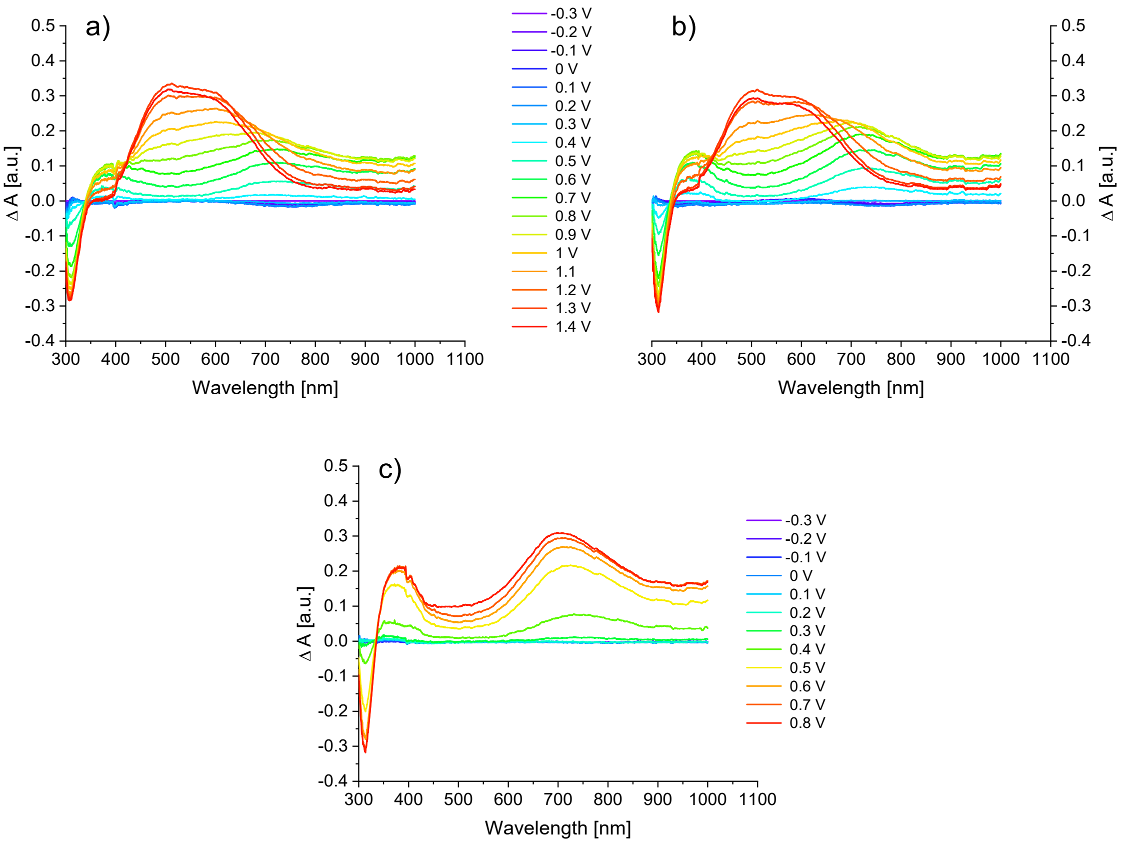

Figure S7. UV-vis spectra of a poly-[Cu(3-MeOSal-Sal)en] film at an ITO-coated glass electrode acquired during redox switching in 0.1 mol dm^{-3} Et_4NBF_4/CH_3CN: **polymers 1** (a), **polymers 3** (b), **polymers 2** (c), referenced to the spectrum of the polymer at -0.3 V.

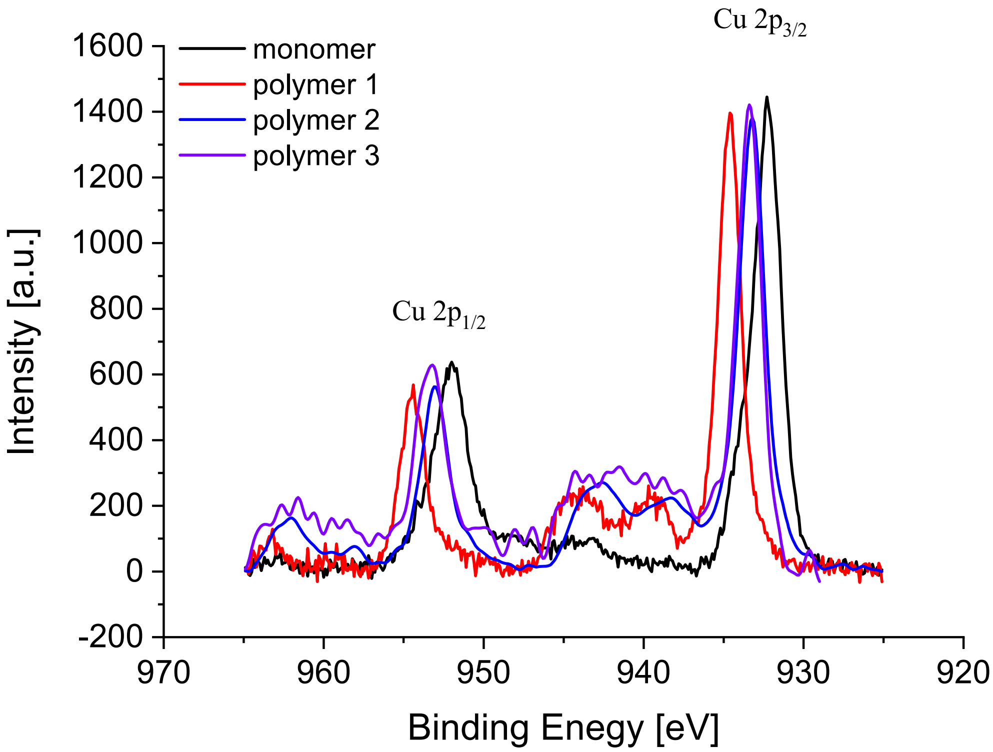

Figure S8. Cu2p XPS spectra of the polymer films.

Scheme S1 (a) shows the electronic structure of the *3d* orbitals of the copper atom. It can be seen from the diagram that there are three spin-allowed transitions. The $d_{x^2-y^2}$ orbital has the highest energy, it is elongated along the coordinate axis, and it is directed toward the ligand. This orbital is most susceptible to destabilization, it is exposed to the field of ligands. The orbital d_{z^2} depends on the environment, as it lies in the xy plane. If the complex is coordinated along the z axis, then the orbitals d_{z^2}, d_{xy} and d_{yz} will be destabilized, their levels will increase, and the transition to $d_{x^2-y^2}$ will require less energy. An electron poor ligand can lower the energy of the highest occupied molecular orbital and thus stabilize the Cu(III) state.

Oxidation of such systems can give one of three electronic states: 1) complex $3d^8$ Cu(III)–Salen (S=0), diamagnetic [4,5]; 2) Cu(II) complex antiferromagnetically bound to a phenoxyl radical (S=0), such a complex has disturbed symmetry, so we exclude it from further consideration (taking into account the orthogonality between the metallic $3d_{x^2-y^2}$ and the phenolate-based orbital, the antiferromagnetic Cu(II) radical form can be excluded). 3) Cu(II) ferromagnetically bonded to the phenoxy radical (S=1), paramagnetic. Quantum chemical calculations using the TDFT method showed that the most preferred state is 1, since it has the lowest energy [6]. The energy of the highest occupied molecular orbital (HOMO) on the ligand decreases and, thus, the Cu(III) state stabilizes. The authors of [7] also exclude the antiferromagnetic Cu(II) radical form and prefer the ground triplet state. In poly-[Cu(3-MeOSal–Sal)en] both states can be realized, and it is possible that it is in equilibrium and depends on conditions. The environment of the central atom also affects the geometry of the molecule and thus can shift the equilibrium.

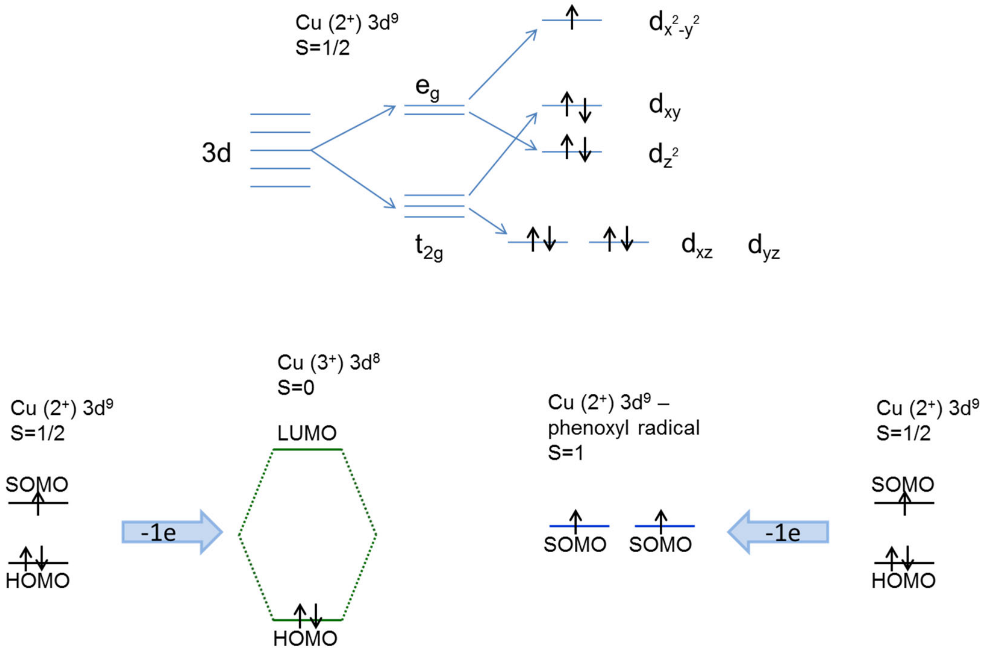

Scheme S1. Схематичное представление HOMO-LUMO energy gap.

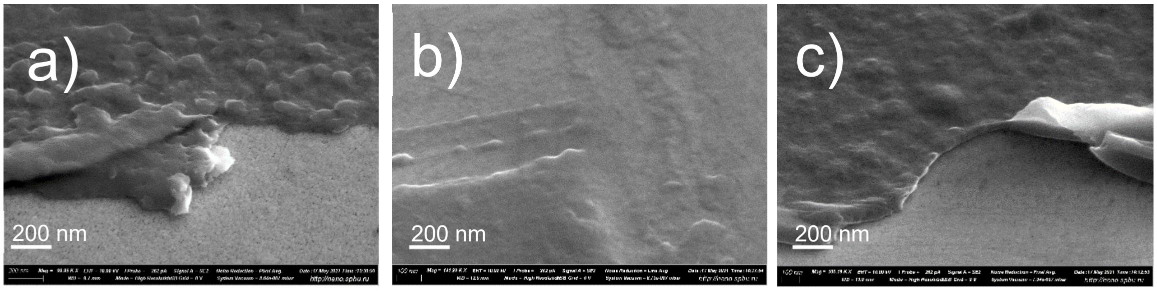

Figure S9. Scanning electron micrographs of poly-[Cu(3-MeOSal–Sal)en] films deposited on a Pt electrode: **polymer 1** (a), **polymer 2** (b), **polymer 3** (c).

References

1. Yankin, A.N.; Lukyanov, D.A.; Beletskii, E. v.; Bakulina, O.Yu.; Vlasov, P.S.; Levin, O. v. Aryl-Aryl Coupling of Salicylic Aldehydes through Oxidative CH-Activation in Nickel Salen Derivatives. *ChemistrySelect* **2019**, *4*, 8886–8890, doi:10.1002/slct.201902385.
2. Laviron, E. General Expression of the Linear Potential Sweep Voltammogram in the Case of Diffusionless Electrochemical Systems. *J Electroanal Chem Interfacial Electrochem* **1979**, *101*, 19–28, doi:10.1016/S0022-0728(79)80075-3.
3. Sterby, M.; Emanuelsson, R.; Huang, X.; Gogoll, A.; Strømme, M.; Sjödin, M. Characterization of PEDOT-Quinone Conducting Redox Polymers for Water Based Secondary Batteries. *Electrochim Acta* **2017**, *235*, 356–364, doi:10.1016/j.electacta.2017.03.068.
4. Storr, T.; Verma, P.; Pratt, R.C.; Wasinger, E.C.; Shimazaki, Y.; Stack, T.D.P. Defining the Electronic and Geometric Structure of One-Electron Oxidized Copper−Bis-Phenoxide Complexes. *J Am Chem Soc* **2008**, *130*, 15448–15459, doi:10.1021/ja804339m.
5. Asami, K.; Tsukidate, K.; Iwatsuki, S.; Tani, F.; Karasawa, S.; Chiang, L.; Storr, T.; Thomas, F.; Shimazaki, Y. New Insights into the Electronic Structure and Reactivity of One-Electron Oxidized Copper(II)-(Disalicylidene)Diamine Complexes. *Inorg Chem* **2012**, *51*, 12450–12461, doi:10.1021/ic3018503.
6. Chiang, L.; Herasymchuk, K.; Thomas, F.; Storr, T. Influence of Electron-Withdrawing Substituents on the Electronic Structure of Oxidized Ni and Cu Salen Complexes. *Inorg Chem* **2015**, *54*, 5970–5980, doi:10.1021/acs.inorgchem.5b00783.
7. Eckshtain-Levi, M.; Lavi, R.; Arora, H.; Orio, M.; Benisvy, L. Tuning the Locus of Oxidation in Cu-Diamido-Diphenoxo Complexes: From Cu(III) to Cu(II)-Phenoxyl Radical. *Inorganica Chim Acta* **2018**, *481*, 143–150, doi:10.1016/j.ica.2017.09.049.
